# A New, Adaptable, Optical High-Resolution 3-Axis Sensor

**DOI:** 10.3390/s17020254

**Published:** 2017-01-27

**Authors:** Niels Buchhold, Christian Baumgartner

**Affiliations:** Institute for Health Care Engineering, Graz University of Technology, Stremayrgasse 16, 8010 Graz, Austria

**Keywords:** tactile sensors, assistive technologies, power wheelchair, medical systems, robotic, joystick, optical sensor

## Abstract

This article presents a new optical, multi-functional, high-resolution 3-axis sensor which serves to navigate and can, for example, replace standard joysticks in medical devices such as electric wheelchairs, surgical robots or medical diagnosis devices. A light source, e.g., a laser diode, is affixed to a movable axis and projects a random geometric shape on an image sensor (CMOS or CCD). The downstream microcontroller’s software identifies the geometric shape’s center, distortion and size, and then calculates *x*, *y*, and *z* coordinates, which can be processed in attached devices. Depending on the image sensor in use (e.g., 6.41 megapixels), the 3-axis sensor features a resolution of 1544 digits from right to left and 1038 digits up and down. Through interpolation, these values rise by a factor of 100. A unique feature is the exact reproducibility (deflection to coordinates) and its precise ability to return to its neutral position. Moreover, optical signal processing provides a high level of protection against electromagnetic and radio frequency interference. The sensor is adaptive and adjustable to fit a user’s range of motion (stroke and force). This recommendation aims to optimize sensor systems such as joysticks in medical devices in terms of safety, ease of use, and adaptability.

## 1. Introduction

The use of sensors as an interface between people and machines is becoming increasingly important in our society. Joysticks can be operated practically in an intuitive manner and are found more and more often in a variety of controller systems as input devices. Areas of application such as medical technology require a high level of safety during use. Moreover, a wide spectrum is necessary for individual users. In particular, for people with physical disabilities such as quadriplegia, spasticity, and muscular dystrophy, using a sensor (joystick) is often a problem since the range of motion in terms of force and stroke undergoes constant changes as a result of such illnesses [1]. Depending on the specifications, off-the-shelf joysticks have a pre-determined accuracy (resolution); a certain amount of force is required to deflect it, and a certain stroke to overcome the necessary paths either by a fixed or very limited amount. Adapting the sensor to one individual is expensive and in some cases impossible when the user’s range of motion and strength is affected by changes in temperature [2]. Standard joysticks are generally two-dimensional control systems for the *x* and *y* directions. In order to steer in the *z* direction, the controller is turned or an additional control element is required. Physically disabled people with spinal cord injury (SCI) [3] usually do not have the fine motor ability required to carry out such an action. A simple push along the *z*-axis is more likely to be possible. The sensor presented here can adapt to the desired strength and range of motion. Thus, there is no need for costly adjustments for individual users, thereby saving significant costs. There are currently 250,000 people living in the United States today with spinal injuries. A total of 47% of them are injured between C1 and C7 in the cervical spine area, rendering them quadriplegic. Each year, 11,000 people suffer a spinal injury [4,5]. In addition to this, there are muscular diseases such as muscular dystrophy. This disease affects between 1 in 3500 to 6000 male babies per year in the United States [6]. These diseases usually require a custom input device in order to enable users to operate electric wheelchairs or computers. However, the sensor can also be adapted to healthy users in the areas of diagnostic devices and surgical robotics, thus making it possible to achieve a higher level of user safety and precision while work is performed. If, for example, a powered wheelchair is used, new control systems such as eye tracking, voice control, and brain-computer interfaces tend to be problematic [7,8,9,10,11]. These types of input methods make it difficult to perform precise and complex control actions simultaneously. Voice controls can usually only process one command at a time and are unreliable in loud environments [12]. The exponential progression of the spring force and the very limited movement of the sensor axis generates a natural force feedback [13]. The user thus receives physical feedback pertaining to the deflection movement. The introduced sensor’s potential areas of application can be expanded without limits. In addition to medical applications, fields such as the automotive, aeronautical, aerospace, marine, and military sectors in particular are conceivable here.

**Figure 1:**
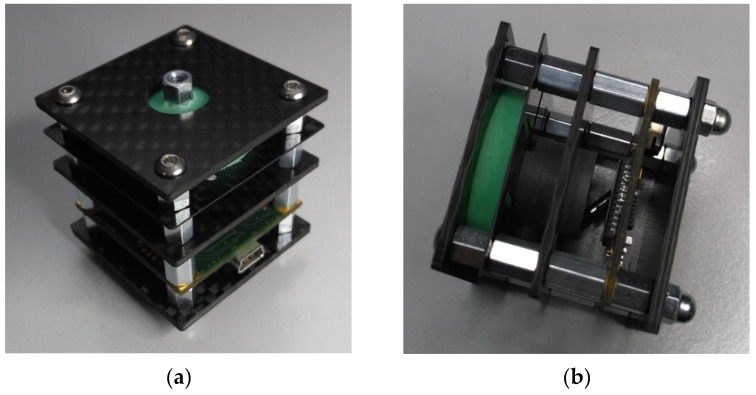
The 3-axis sensor version 3 prototype V12.2 carbon fiber-reinforced polymer (CFRP), (**a**) front view (**b**) side view.

Optical methods for determining positions are well known and are described in the following selected patents and publications [14,15,16,17]. However, this paper introduces the prototype’s hardware (see Figure 1) of a new optical, multi-functional, high-resolution 3-axis sensor and presents the sensor’s novel features and components, and its methods of positioning. These include:

1. The specific construction of the projector unit
2. The minimal distance between projector unit and image sensor
3. The construction and formation of the movement carrier made of carbon fiber-reinforced polymer (CFRP)
4. The movement carrier’s ability to return to its exact position of rest after deflection
5. Adherence to standard construction dimensions and the potential to establish the sensor in various areas of application
6. The algorithm to customize the device to changed clinical symptoms
7. The use of a software to increase frames per second (fps) to 150, which increases user safety and sensor responsiveness
8. Testing plausibility of projections to increase user safety
9. Using the natural force of feedback effect due to its construction
10. The increase in usable resolution by a factor of 100 through interpolation of the projected image using grey values and limits

The applied optical approach substantially increases safety of use. Disruptive factors such as electromagnetic (EMI) and radio frequency interference (RFI) or differences in temperature have a marginal effect on the sensor if at all. Because the sensor can be manufactured very inexpensively, the consumer area is also an interesting option. With its very short axis movement of <1°, the operation and reaction time is substantially higher than that of off-the-shelf joysticks. The sensor is completely removable from its casing on the input side, making sterilization or disinfection very easy. The joystick also operates without a rubber boot. The sensor is, for the most part, free from wear and tear and can be placed in a waterproof casing.

## 2. Hardware of the 3-Axis Sensor

### 2.1. Basic Construction

**Figure 2:**
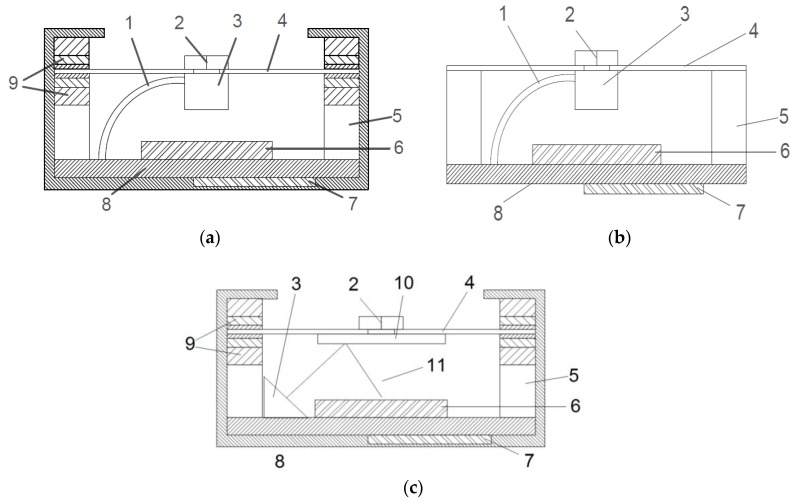
(**a**) The 3-axis sensor, version 1; (**b**) The 3-axis sensor, version 2; (**c**) The 3-axis sensor, version 4.

The sensor consists of a force transducer 2 and a projection unit 3, which is firmly affixed to a movement carrier 4 (Figure 2). An image sensor is positioned underneath the projection unit at a set spacing. The angle of the movement carrier to the image sensor changes as a result of force applied to the force transducer. In doing so, the position of the projection on the image sensor shifts. Based on the position, size, and distortion of the projection, the current *x*, *y*, and *z* coordinates are calculated. This happens in relationship to the degree the angle changes and the pressure on the axis. In order to exchange the sensor for a commercially available joystick without further constructive problems, [18] standard designs have been adhered to during development. The 3-axis sensor casing measures (Length × Width × Height) 40 mm × 40 mm × 37 mm. Depending on the software configuration, the connection cables, which are located on the side or at the rear, can provide digital and/or analog data.

The 3-axis sensor consists of the following components:

1. power supply line, projection unit
2. force transducer
3. projection unit
4. movement carrier
5. spacer
6. image sensor
7. microcontroller
8. circuit board
9. polymer sandwich
10. mirror
11. laser beam

### 2.2. Projection Unit and Image Sensor

**Figure 3:**
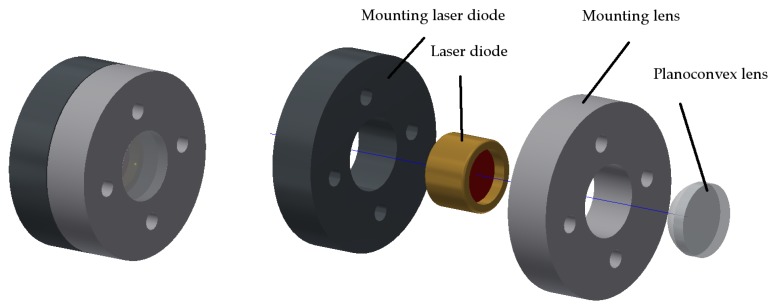
Left projection unit drawing; right projection unit exploded view drawing.

In its simplest construction, the projection unit consists of a laser diode with a downstream plano-convex lens [19]. This configuration can also be substituted by a collimator lens [19] and a shape template. The image sensor is positioned underneath the projection unit at a set spacing. Depending on the application and the image sensor that is used, frame rates up to 150 fps can be achieved. The sensor’s maximum resolution depends on the image sensor type. For example, when using a ½ inch sensor with 1280 (horizontal) × 1024 (vertical) pixels, SXGA/1.3 MP [20], without further calculation, 1280/2 = 640 digits (horizontal) and 1024/2 = 512 digits (vertical) for each direction can be achieved. The structure of the projection unit is shown in Figure 3.

The projection unit has a diameter of 17 mm and a height of 10 mm. The relatively large diameter has no influence on the housing size. Since the movement carrier and the projection unit are to be connected to one another in a torsion-free manner, the supporting surface is necessary. With this structure, it is possible to place the focal point at a distance of 10 mm in front of the light output of the laser diode. When sub-pixels (gray tones) are included in the calculation and the resulting pool of light is interpolated, at least two decimal places can be added. The resolution is thus increased 100-fold with a resolution of 64,000 (H) und 51,200 (V) available in each direction. When using an off-the-shelf 18.1 MP sensor with 4912 (H) × 3684 (V) pixels [21], based on the calculation mentioned above, there is a resolution of 245.600 (H) × 184.200 (V) digits in each direction. These values should be sufficient even for highly sensitive measurements. In the consumer sector (personal computer joystick substitute), resolutions of 0.36 MP and frame rates of 30 fps are totally sufficient.

### 2.3. Different Versions

**Figure 4:**
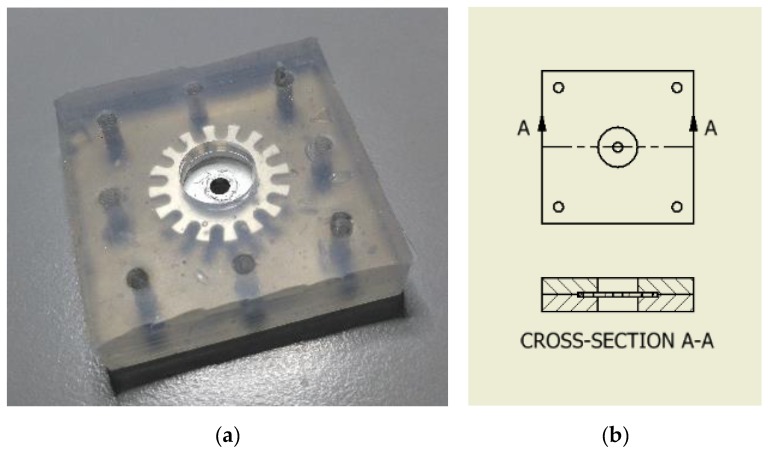
(**a**) Polymer with encapsulated movement carrier, encapsulated version 1 (**b**) Polymer with movement carrier, cross-section, version 1.

Depending on the purpose of use, the sensor can be operated in different variations:

Version 1 (see Figure 2a): The movement carrier is positioned in a polymer sandwich and consists of a rigid material such as steel or carbon fiber-reinforced polymer (CFRP). Through the force picked up by the force transducer, the movement carrier tilts. The degree of tilt depends on the force being applied and the polymer’s shore hardness (Figure 4).

**Figure 5:**
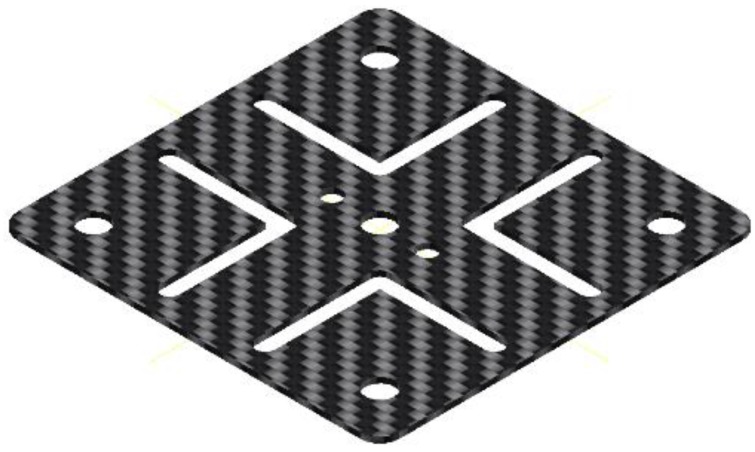
CFRP movement carrier, version 3.

Version 2 (see Figure 2b): The movement carrier is manufactured out of elastic material (e.g., CFRP in various strengths from 0.45 mm to 2 mm). The movement carrier is rigidly affixed to the casing and withstands bending and torsion. The cutout illustrated in Figure 5 is conducive to the movement carrier’s deformation behavior.

Version 3: The same as Version 2 but includes an additional polymer cushion above the movement carrier.

Version 4 (see Figure 2c): The projection unit, which produces images, is replaced by a mirror. In order to generate a projection on the image sensor, the projection unit is affixed to the circuit board next to the image sensor and projects back over the mirror to the image sensor. The movement carrier’s changed angle causes the projection to reposition according to the forces acting on the projection.

### 2.4. Movement Carrier

The movement carrier is selected depending on the application and the desired input force spectrum. In version 1, a rigid material such as steel (diameter > 1 mm) or CFRP (diameter > 1.5 mm) is integrated. In versions 2 and 3, flexible materials such as CFRP (0.3–1.1 mm) are used. The materials used in version 2 and 3 should be characterized by excellent spring behavior, which is why CFRP is predestined for this application. The pivot point of the bearing is located at the height of the movement carrier. The distance between the movement carrier and the image sensor surface is 17 mm. For a diagonal of the image sensor of 1/2 inch (12.7 mm) and a resolution of 1.3 megapixels, the pixel size is 5.3 μm. The angular deviation of ~17.86° × 10^−6^ can be calculated from the formula x=r sin(φ), indicating the deflection of the laser beam by one pixel on the image sensor. The material and the shaping of the movement carrier were chosen such that after a deflection of the laser beam over the entire sensor surface, the resting position is found, with a drift of ±2 digits. Temperature influences of −30° to 80° Celsius produce an additional temperature drift of ±1 digit. Thus a maximum drift of ±3 digits is possible. The maximum angular writing of the laser beam then amounts to ±53.58° × 10^−6^. In order to intercept this drift, a neutral window of ±5 digits is taken into account by the software. The output signals are not changed until the calculated coordinates are outside the described neutral window. The technical design consists of two layers of CFRP fabric which were installed in a unidirectional manner at 0°/90° to one another. The resin content is 35%. After 250,000 maximum deflections, the movement carrier’s drift of ±2 digits did not change. Further tests will follow.

### 2.5. Polymer Sandwich

The movement carrier is positioned in a polymer sandwich in version 1. The movement carrier’s spring is achieved through a particular arrangement of polymer layers. As a result, the Shore hardness increases (transition from soft to hard). Owing to the polymer arrangement (position 9) shown in Figure 2, the movement carrier is capable of absorbing a large spectrum of forces upon it (approximately 1 g to 5 kg). Due to the individual layer’s varying degrees of Shore hardness, each becomes active under different forces. Once a layer’s maximum compression is reached, the layer with the next degree of hardness becomes active and takes on the movement until it too reaches its maximum compression and so on.

## 3. Sensor Operations

### 3.1. Fundamentals

**Figure 6:**
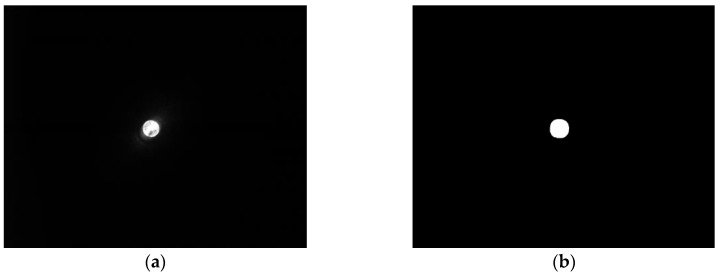
(**a**) Complete frame without image processing; (**b**) Complete frame with image processing to binary mode.

When forces act on the movement carrier’s force transducer, the movement carrier changes its tilt and/or the spacing between it and the image sensor below. In doing so, the projection unit, which is firmly affixed to the movement carrier, generates an image of a filled circle on the image sensor (see Figure 6a for full view of a 1.3 MP image sensor without photo editing; Figure 6b shows a full view of the image sensor in binary mode).

**Figure 7:**
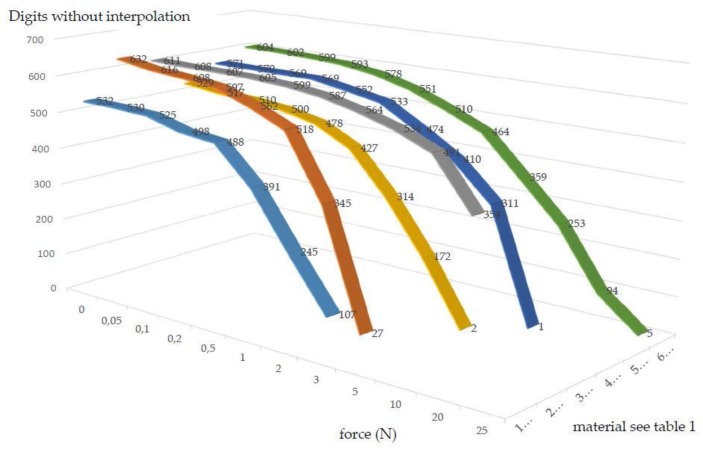
Visualized, measured values from Table 1.

The movement carrier’s restoring force occurs either through the arrangement of the polymer layers in version 1 or the resilience of the material used as in version 2 and version 3. Table 1 and Figure 7 show the deflection relative to the applied force. Different polymer arrangements and types are shown here as examples. Due to the fact that the measurement curves are identical in both directions, the measurement was carried out for one direction only. Force was applied by a 45-mm lever. In doing so, relatively soft polymer layers were used and a force up to 25 N was measured. “out” means that the light circuit has left the active sensor field.

### 3.2. Projection, Image Analysis and Data Output

#### 3.2.1. Projection

**Figure 8:**
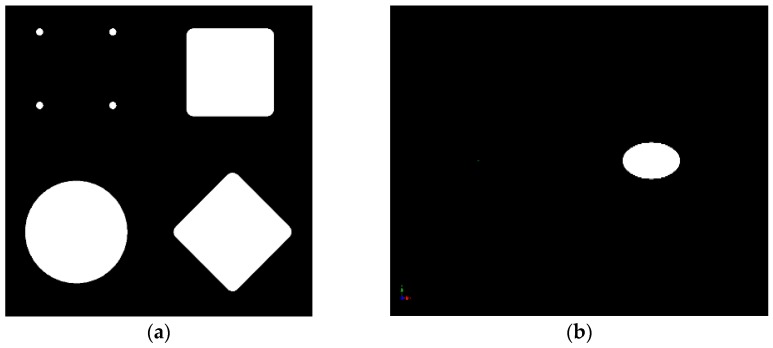

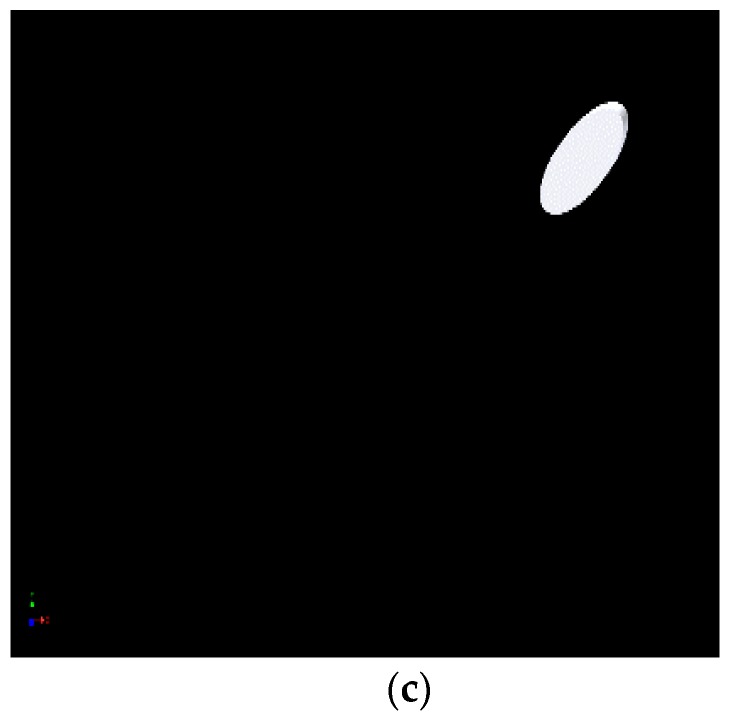
(**a**) Various projection images schematically; (**b**) Deflection of the circle (Figure 6b) on the *x*-axis schematically; (**c**) Deflection of the circle (Figure 6b) on the *x*- and *y*-axis schematically.

As shown in Figure 8, geometric shapes of all kinds can be used for the projection (Figure 8a–c are shown in schematic form).

As an example, the simplest geometric shape (full circle; see Figure 8a on bottom left) was used for the prototype presented here. By using four dots (see Figure 8a top left) more than three axes can be measured. By measuring the four points with respect to each other as well as the measurement within the image sensor coordinate system, the change can be detected along any axis. Unfortunately, the measurement of the four points is very computation-intensive, which reduces the framerate of the prototype to about 12 fps. Faster processors can improve this. Figure 6 shows the resting state. As a result of the manufacturing allowances, it does not matter whether the projection of the circle is exactly in the middle of the image sensor or slightly off-center. The absolute zero position, or resting position, is set during calibration. The calibration process usually takes place only once after production. This avoids higher production costs since the manufacturing allowances are relatively insignificant. Figure 8b shows the movement carrier in the *x* direction. A distorted ellipsis is produced due to the angle of the light. In addition, the diagonal deflections in the *x* and *y* directions are shown in Figure 8c.

#### 3.2.2. Image Analysis and Data Output

**Figure 9:**
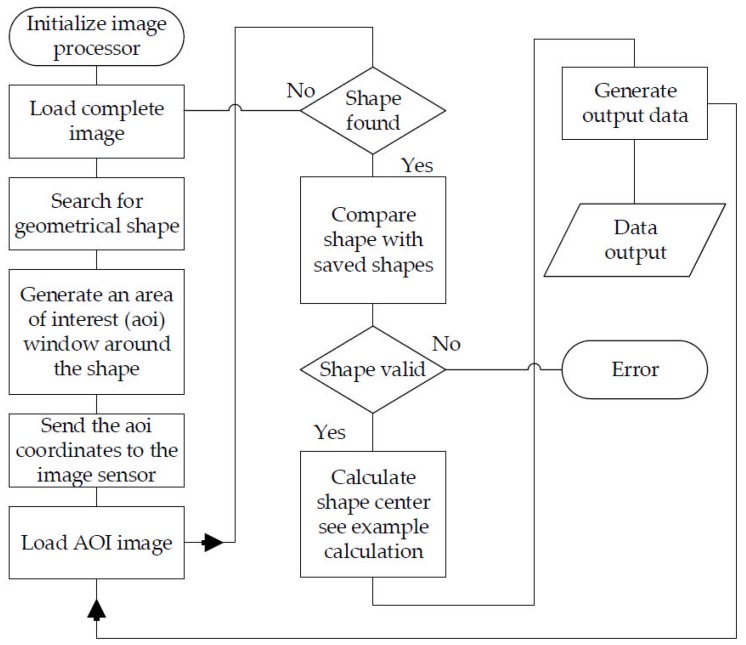
Schematic software procedure—normal use.

The image analysis software (Figure 9) first searches for the previously defined shape on the captured image. In order to reduce computation time, once the image is found, only the area of interest [22] around the object is examined. This approach increases the processing time substantially. If the image is no longer found in the scanned image area, the entire image is scanned again. The sub-pixels’ brightness values are then analyzed and the center of the projection is calculated through interpolation. This increases the accuracy by two decimal places.

**Figure 10:**
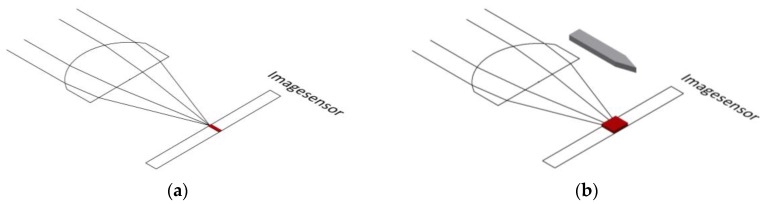
(**a**) Focusing without pressed *z*-axis; (**b**) Focusing with pressed *z*-axis.

A button must be pressed in order to start the learning process for the individual adjustment. The microprocessor then records the maximum achieved coordinates in each direction. In addition, the distortion of the geometric figure is stored along with the maximum achieved coordinates. If it is a circle, the maximum *x*- and *y*-dimension of the figure is stored. In normal operation (Figure 9), the shape found is compared with the stored shapes and their coordinates. If the figure does not correspond to the expected figure, an error is generated to protect subsequent systems from malfunctions. The project focus is set in such a way that the focal point is below the image sensor’s surface in a state of rest. As a result, a movement in the *z* direction removes the focal point from the image sensor or brings it closer. The projected circle thus changes in size depending on the force being applied (Figure 10a, focusing without pressed *z*-axis). A pull of the *z*-axis decreases the size of the projection, while a push of the *z*-axis enlarges the projection (see Figure 10b).

**Figure 11:**
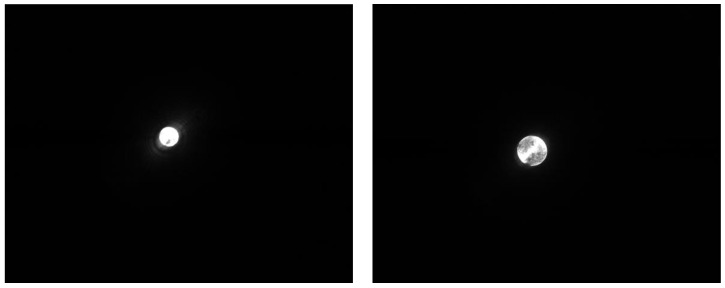
Difference between neutral *z*-axis (**left**) and pressed *z*-axis (**right**).

The image analysis software detects either the smaller circle of light or the larger circle of light and then calculates the current *z*-axis coordinates (see Figure 11). These highly accurate coordinates are then passed on to the downstream system. While doing this, serial values or different bus protocols (CAN, SPI, I2C, analog voltage, etc. in the next step of development) can be generated.

### 3.3. Plausibility Check

The sensor should also be utilized in highly sensitive or safety-related areas. To do so, a plausibility check is performed. The projected shape is naturally distorted during the projection (Figure 8b,c). When the projected circle moves away from the middle of the image sensor, the circle becomes an ellipsis. Since an ellipsis has two focal points F1 and F2, the resulting spacing is calculated. The spacing of the focal points increases the further the movement carrier is moved. In order to identify the maximum allowable spacing between F1 and F2, the movement carrier is moved in each direction once with the maximum amount of force permitted. Ideally, it should be a circular movement with the maximum force permitted. This circular movement can be performed either manually or with a calibration device. During the calibration movement, the maximum distortions and their positions are extracted and saved permanently. All of the detected projections are compared to the previously saved images during normal operation. When a maximum position or a maximum distortion is exceeded, measures can be taken to protect the downstream system from malfunctions. Additionally, other special cases can be identified. In the event that the projection unit leaves its position of rest due to a hardware defect without being moved, the resulting projection would be distortion-free in another position. This is considered an error because it is not possible to detect a distortion-free projection at other coordinates such as the original position of rest. The plausibility check can be expanded depending on the performance of the image sensor and the microcontroller that are in use. In addition, motion patterns or speeds can be analyzed and checked for plausibility.

### 3.4. Individual Adjustment of the 3-Axis Sensor

Due to the various symptoms of individual medical conditions, different input forces and input lifts are required. Depending on the specifications, standard joysticks have a pre-determined accuracy (resolution), a certain amount of force is required to deflect it, and a certain stroke to overcome the necessary paths by a fixed amount. As described in the introduction, certain groups of physically disabled people are unable to use standardized joysticks. The issue has to do with this group of individuals’ range of motion in terms of force and stroke. Moreover, the existing range of motion is affected by outside influences such as the ambient temperature [2]. A permanent readjustment of the sensor would be necessary for long-term use. In order to adapt the sensor to the patient’s motion and stroke movement abilities, the user moves the 3-axis sensor at least once in each direction with a circular movement. The *z*-axis can also be configured through vertical pressure and tension applied to the movement carrier. The maxima of the *x*-, *y*-, and *z*-coordinates are then saved.

**Figure 12:**
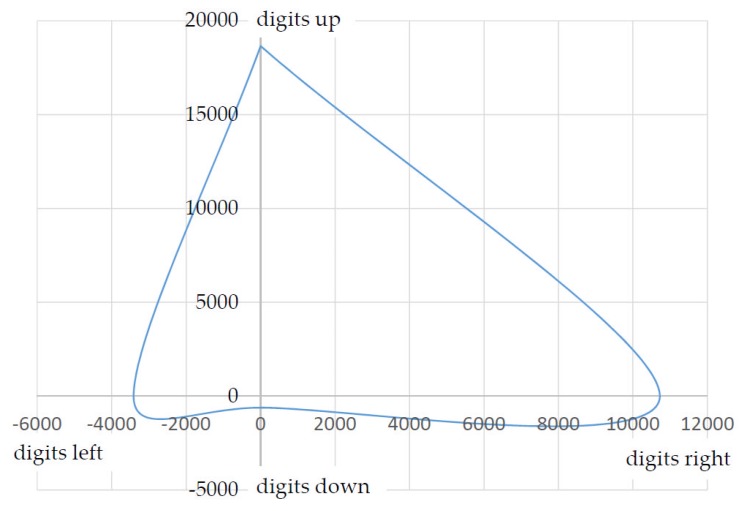
Force curve (force to digit); interpolated values.

As an example, the force or the stroke of a patient suffering from muscular disease was documented. The result in Figure 12 shows an inhomogeneous progression of force applied in different directions.

When a reference was established (deflection and force to digit), Figure 12 can be used to directly determine the force and stroke the patient applied. The relationship between stroke and the change in coordinates is of course just as dependent on the length of the lever used. In a test case, the patient was able to carry out the following deflection, i.e. force (the *z*-axis is not taken into account here; Table 2).

These values cannot be transmitted directly to a power wheelchair. The patient would simply drive the wheelchair forwards and to the right while traveling at a sufficient speed. The values achieved for reverse and left are not sufficient to move the wheelchair. Suitable factors or divisors can provide the desired consistent output signal. Example: Common output values are in the 10-bit range ((measured value/1024) 2 = divisor; Table 3).

All of the coordinates documented during use for this user, then have to be converted with the divisor to attain a consistent output signal.

**Figure 13:**
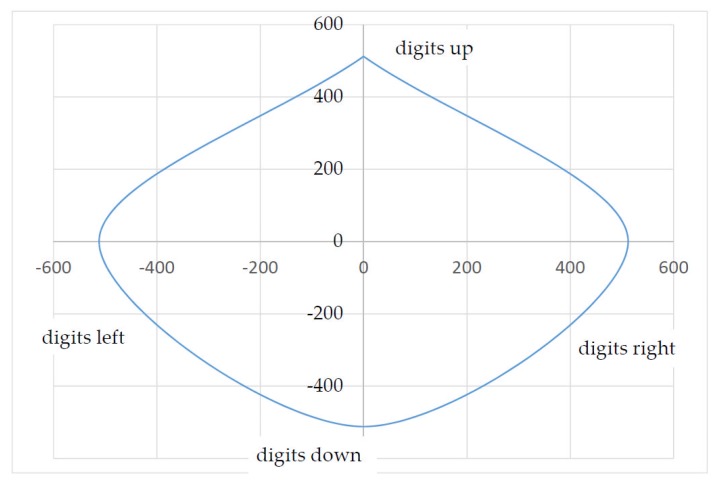
Output data curve.

Example calculation:

All values are multiplied by 100 by the interpolation of the image (right value, see Table 2; 10-bit output signal; image sensor 1280 × 1024).
$X_{MaxRes}=128.000\;digits;$Maximum resolution in horizontal direction;$X_{MaxResRight}=\frac{X_{MaxHorRes}}{2};$
$X_{MaxResRight}=64.000\;digits;$Half resolution, each direction;$X_{Neutral}=63.891\;digits;$Measured neutral value;$X_{NeutHysteresis}=500\;digits;$Hysteresis for a neutral window in the middle position;$X_{MaxRight}=74.615\;digits;$Learned maximum value to the right;$X_{AbsolutMaxRight}=X_{MaxRightPat.1}-X_{Neutral};$
$X_{AbsolutMaxRight}=10.724\;digits;$Absolute maximum value to the right;$Resolution_{OutMax10bit}=1024\;digits;$Desired maximum output value;$Resolution_{EachDirection}=\frac{Resolution_{OutMax10bit}}{2}$
$Resolution_{EachDirection}=512\;digits;$Desired maximum output value, each direction;$X_{DiviRight}=\frac{X_{AbsolutMaxRight}}{512\;\mathrm{digits}};$
$X_{DiviRight}=20,95;$Calculated divisor;$X_{RightExample}=4.200\;digits;$Example value to the right;$X_{OutRight}=\frac{X_{RightExample}}{20,95};$
$X_{OutRight}=200\;digits$Digital output value;

By means of the algorithm shown above, the coordinates of the inhomogeneous input forces (see Figure 12) are converted into homogeneous output data (see Figure 13). The user can then, for example, move a computer mouse or an EPW (electric power wheelchair) at the same speed in all directions independently of its inhomogeneous force-lifting ratio. The output values calculated by the divisor are denoted in Table 4 and visualized in Figure 13.

### 3.5. Hardware

**Figure 14:**
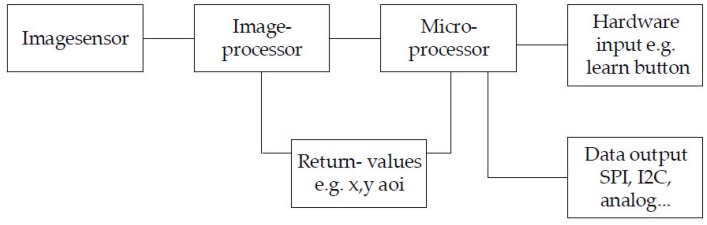
Schematic sensor hardware.

The hardware (see Figure 14) consists of the image sensor, the image processor and a further microprocessor. The microprocessor first configures the image processor and then receives image data for further processing (see Section 3.2.2). In order to increase the processing speed, *x*, *y* coordinates for the relevant image excerpt (AOI) are transferred to the image processor. The microprocessor generates the desired output data and obtains the learning command via an I/O port.

## 4. Results and Discussion

The sensor described here is in the prototype stage. Due to the optical implementation, it should be resistant to outside interference such as EMI or RFI. In version 1, hot, vulcanizing 2 components silicone rubber is used, which has optimal spring properties. Unfortunately, this ability is not sufficient to achieve a satisfactory result. Depending on the axis deflection, the neutral position is more or less difficult to reach. This circumstance can be compensated for because the neutral values can be reproduced exactly. In versions 2 and 4, the movement carrier is made of CFRP and firmly affixed to the casing. Compared to fiber-reinforced polymer (AFRP) and glass-fiber reinforced polymer (GFRP), CFRP has superb dynamic properties. The dynamic damping capacity of AFRP under a dynamic load is six times higher than GFRP and nine times higher than CFRP [23]. Using CFRP solves the problem described above and the spring precision is at ±2 digits (without interpolation, image sensor 1.3 MP). The climate chamber was successfully completed (−30 degrees to 80 degrees). The deviation was at ±1 digit (without interpolation, image sensor 1.3 MP). The first tests under laboratory conditions were successfully completed. During the tests, the 3-axis sensor was used in place of a mouse and to operate a power wheelchair [24]. As long as a physically disabled patient still has some type of physical capability (hand, finger, foot, toe, head, chin, etc.), it should be possible to use this sensor. Owing to the sensor’s ability to learn, there are no additional costs. If the clinical symptoms change for the worse, the sensor can be adapted to the new conditions immediately. Thanks to the high resistance to interference, there is potential for application to areas other than the medical sector such as the automotive, aeronautics, aerospace, marine and military fields. Owing to its resolution and reproducibility (movement to digits), geophysical sensors could also be replaced by this sensor. A screwed-on rod or ball to receive the force can come into resonance oscillation when a defined oscillation frequency acts on the sensor. Thus, the system can begin to “vibrate”. This particular case can also occur in off-the-shelf joysticks. To compensate, a polymer ring can be installed above the movement carrier (version 3), which functions as a shock absorber and dampens vibrations. For technical reasons, the force to be exerted increases exponentially to the deflection, causing a natural force feedback. This positive effect can be explained as follows: Since the movement carrier and thus also the axis of the sensor can only be moved by <1° in any direction, the user perceives it to be a rigid system. When a rigid system is subjected to an input force, the skin feels a pressure proportional to the applied input force. Users reported a very pleasant effect compared to conventional joysticks. Off-the-shelf joysticks are generally equipped with a reset spring. The effect of a proportional increase of the counterforce is very small. Without seeing a commercially available joystick, the user cannot derive a conclusion from the applied force to the actual deflection. Noise in the image sensor is also another issue. In a state of rest, the coordinates change by up to 2/100 digits (with interpolation, image sensor 1.3 MP). For the most part, this camera noise can be removed on a mathematical basis [22].

## 5. Conclusions

Our results show the feasibility and practicability of the new optical, multi-functional, high-resolution 3-axis sensor. Compared to standard, high-resolution sensors (resistive, inductive, or capacitive) the sensor presented here works on a completely digital basis. No compensation is required to adapt the sensor to fluctuations in temperature, for example. Depending on the intensity of the plausibility check, the software requires a substantial amount of calculation time. In the version shown here, the focus of the calculation can be on maximum frame rate or on maximum resolution. Because the different uses and applications usually only require one focus, the sensor’s software can be optimized for each purpose. The current prototype can achieve a maximum resolution of 60 fps. When using the area of interest (AOI) function, 150 fps can be processed. This problem can be solved by using faster image sensors and faster microcontrollers. In addition, the sensor can also work with more than 3-axes, for example, when a square is used as the projected shape. The image analysis software then also detects a rotary movement of the axis, the computation time however increases substantially.

## Figures and Tables

**Table 1:** Selected measurements with different materials and versions.

Excerpt Test Series/Force (N) 45-mm Lever	0	0.05	0.1	0.2	0.5	1	2	3	5	10	20	25
1 Mat.T13-44T1 V11.0	532	530	525	498	488	391	245	107	out	out	out	out
2 Mat.T13-44T1 V10.2	632	616	608	597	562	518	345	27	out	out	out	out
3 Mat.T11-55 V10.2	611	608	607	605	599	587	564	534	491	354	out	out
4 Mat.T13i-T1a V10.2	529	517	510	500	478	427	314	172	2	out	out	out
5 Mat.T1 V10.2	571	570	569	569	552	533	474	410	311	1	out	out
6 Mat.CFRP 055 V12.2	604	602	599	593	578	551	510	464	359	253	94	5

**Table 2:** Patient measurements and applied force.

Direction To	Maximum Digits	Approximate Force
forward	18.653	3.92 N
back	611	0.07 N
left	3411	0.74 N
right	10.724	2.12 N

**Table 3:** Patient measurements and calculated divisor.

Direction To	Maximum Digits	Divisor for Each Direction
forward	18.653	36,43
back	611	1,19
left	3411	6,66
right	10.724	20,95

**Table 4:** Maximum patient measurements and calculated output values.

Direction To	Maximum Digits	Divisor for Each Direction	Calculated Output Values
forward	18.653	36,43	512
back	611	1,193	512
left	3411	6,66	512
right	10.724	20,945	512
